# Mineralocorticoid Effects in Cushing’s Disease: A Case Report

**DOI:** 10.7759/cureus.75780

**Published:** 2024-12-16

**Authors:** Vânia Rodrigues Pereira, Beatriz Castro Silva, Daniel Castanheira, Gabriel Ferreira, Raquel Almeida

**Affiliations:** 1 Internal Medicine, Hospital Beatriz Ângelo, Unidade Local de Saúde de Loures/Odivelas, Loures, PRT

**Keywords:** cushing's disease, hypokalaemia, pituitary macroadenoma, refractory hypertension, secundary hypertension

## Abstract

Cushing’s syndrome is a rare disease caused due to prolonged exposure to excess glucocorticoids. Although rare, diagnosing Cushing’s syndrome is clinically significant as it allows tailored and timely management and significant reduction or even prevention of the comorbidities caused by cortisol excess. This report delineates the presentation of a 44-year-old female with refractory secondary hypertension and severe hypokalaemia, initially thought to be caused by hyperaldosteronism. Upon a more careful physical examination, the presence of moon facies, acanthosis nigricans and violaceous abdominal striae raised suspicion of hypercortisolism. Laboratory tests confirmed the suspicion with increased cortisol and adrenocorticotropic hormone (ACTH) levels. Furthermore, imaging findings led to the final diagnosis of Cushing’s disease due to an ACTH-secreting pituitary macroadenoma. The patient underwent successful transsphenoidal surgery, resulting in substantial clinical improvement, evidenced by significant weight loss and hypertension with decreased need for drugs. When left untreated, patients with Cushing’s disease have a higher mortality rate than the general population. This case underscores the critical importance of keeping in mind secondary endocrine causes in the context of resistant hypertension especially with complex metabolic disturbances and recognizing the most characteristic features of this disease.

## Introduction

The most common cause of supraphysiological levels of cortisol is the exogenous administration of glucocorticoids, causing an iatrogenic Cushing’s syndrome. According to population-based studies, the annual incidence of Cushing’s disease is two to three cases per million people [1]. Cushing’s syndrome is traditionally classified as adrenocorticotropic hormone (ACTH) dependent or ACTH independent. ACTH-dependent Cushing’s syndrome represents 80%-85% of the cases, and of these, Cushing’s disease is the most frequent (68%). Understanding this distribution, and that majority of the cases are ACTH dependent, helps clinicians focus on assessing ACTH levels when hypercortisolism is suspected. Within this category, Cushing’s disease being the most prevalent makes a pituitary source the primary consideration, with diagnostic tests such as pituitary MRI often being the first imaging study performed. ACTH ectopic production accounts for 12% of cases and should be considered when symptoms progress rapidly or are accompanied by signs of malignancy. The extremely rare occurrence of corticotropin-releasing hormone (CRH)-producing tumours (<1%) should only be factored in after more common causes have been excluded. For the remaining 15%-20% of cases that are ACTH-independent, suppressed ACTH levels point to adrenal pathology, such as adrenal adenomas or carcinomas. In this case, the next diagnostic step is imaging of the adrenal glands [1-3]. Understanding these proportions helps clinicians ensure a structured approach to diagnosis, allowing for efficient resource utilization, accurate diagnosis, and timely treatment. This also minimizes unnecessary testing and misdiagnoses, ultimately improving patient outcomes [4].

A thorough clinical history is pivotal in diagnosing and excluding iatrogenic Cushing’s syndrome, as it directly identifies exogenous glucocorticoid exposure, the most common cause of hypercortisolism. By carefully exploring the patient’s medication use, as well as, over-the-counter or alternative treatments, clinicians can pinpoint the source of cortisol excess. This information often provides the definitive diagnosis, avoids confusion with conditions that mimic Cushing’s syndrome and prevents unnecessary and invasive tests. This differentiation is crucial because iatrogenic Cushing’s syndrome is managed by tapering glucocorticoids rather than surgical or medical interventions aimed at endogenous sources [4,5]. The diagnosis of Cushing’s syndrome requires biochemical confirmation through screening tests such as 24-hour urinary free cortisol, late night salivary cortisol or dexamethasone suppression test, with at least two measurements recommended for reliability. Random plasma cortisol levels have no value due to significant diurnal variability [1,4,5]. Subsequent diagnostic tests depend on the suspected aetiology, as previously described.

Cortisol excess disrupts homeostasis, leading to a wide range of signs and symptoms. Albeit none are pathognomonic, some are more discriminatory, including reddish purple striae, plethora, proximal muscle weakness, bruising with no obvious trauma, and unexplained osteoporosis. Although full blown cases of Cushing’s disease may be evident, diagnosis of mild cases can be challenging due to symptom overlap with common conditions such as obesity, diabetes and hypertension [1,2,5,6].

Hypertension in Cushing’s syndrome arises from complex mechanisms, including the activation of mineralocorticoid receptors when cortisol levels exceed the capacity of 11β-hydroxysteroid dehydrogenase type 2 to inactivate cortisol into cortisone, leading to sodium retention, potassium excretion and blood volume expansion, resulting in apparent mineralocorticoid excess and suppressed renin secretion. Additional contributing mechanisms include an imbalance between vasodilatory and vasoconstrictor agents such as endothelin, nitric oxide and prostacyclin, endothelial dysfunction and development of metabolic syndrome [3,6-9]. While addressing cortisol overproduction, direct antihypertensive treatment is necessary to mitigate the cardiovascular risks associated with prolonged high blood pressure. Given the underline pathophysiology, mineralocorticoid receptor antagonists, such as spironolactone or eplerenone, are often first-line agents because they counteract cortisol-induced sodium retention and hypokalaemia. Calcium channel blockers and angiotensin-converting enzyme inhibitors or angiotensin receptor blockers are also commonly used, particularly in patients with comorbidities such as diabetes or heart disease, as they provide additional cardiovascular protection [6].

The therapeutic approach is fundamentally guided by the underlying mechanism of Cushing’s syndrome. In ACTH-dependent cases, the interventions are targeted at suppressing or removing the source of ACTH overproduction. In Cushing’s disease, the preferred treatment is transsphenoidal surgery to remove the pituitary tumour, often curative. If surgery is unsuccessful or contraindicated, alternative treatments such as radiotherapy or cortisol-lowering medications are considered. For ectopic ACTH production, the therapeutic focus shifts to identifying and resecting the responsible tumour, though medical therapy and bilateral adrenalectomy may be necessary in advanced or inoperable cases. ACTH-independent cases require strategies tailored to adrenal-specific pathology. Unilateral adrenalectomy is the primary treatment for cortisol-producing adrenal adenomas. In adrenal carcinomas, aggressive surgical resection, often supplemented with adjuvant therapies is needed. For bilateral adrenal hyperplasia, bilateral adrenalectomy may be required, while milder forms might be managed with medical therapies aimed at reducing cortisol production [9,10]. These therapeutic nuances underscore the importance of precise diagnosis in guiding effective interventions.

This report illustrates an unusual presentation of Cushing’s disease, with hypertension and electrolyte imbalances, more in agreement with mineralocorticoid disturbances. This case also serves as an example of how a condition can go undiagnosed for years; the patient had been hypertensive since the age of 30, which led to the development of preventable comorbidities.

## Case presentation

A 44-year-old woman, previously independent in her daily activities, presented to the emergency department with generalized edema predominantly affecting the lower extremities and facial region. These symptoms were accompanied by profound fatigue, insomnia, increased appetite, and a substantial weight gain of 10 kilograms over two months. Her medical history was notable for class III obesity (BMI 42 kg/m²) and hypertension diagnosed at the age of 30, which was not well-controlled and did not meet target objectives. Her gynaecological history revealed irregular menses with prolonged intermenstrual intervals since the age of 40, and an obstetric history of three pregnancies, including one complicated by preeclampsia requiring induction of labour. Chronic antihypertensive therapy included amlodipine and olmesartan (5 mg/20 mg). Her mother’s early-onset hypertension was the most salient familial antecedent. The presence of an early diagnosis of hypertension, combined with the presenting symptoms and a family history of early-onset hypertension, should raise suspicion for secondary hypertension.

On presentation, her blood pressure was markedly elevated (191/111 mmHg). Pertinent physical findings included non-pitting peripheral edema, "moon" facies, acanthosis nigricans on cervical folds, and violaceous abdominal striae. Laboratory evaluation revealed severe hypokalaemia (2.4 mmol/L) and metabolic alkalosis (pH 7.48, bicarbonate 32 mmol/L, pCO₂ 44 mmHg). Electrocardiographic analysis demonstrated low-voltage QRS complexes and T-wave flattening in precordial leads.

**Figure 1:**
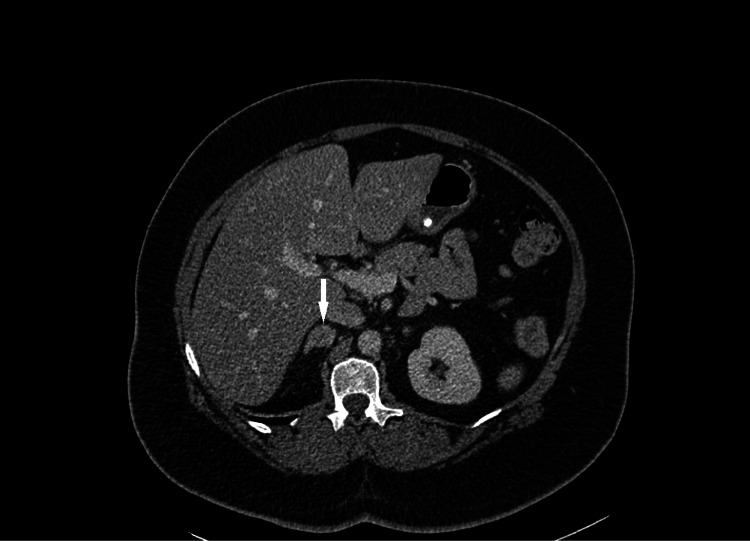
Computed tomography scan of the abdomen showing bilateral adrenal adenomas (right: 2 cm, left: 1.2 cm), with the arrow indicating the right adrenal adenoma

In hyperaldosteronism, aldosterone causes potassium excretion in the distal nephron causing a compensatory shift of hydrogen ions into cells leading to metabolic alkalosis; concurrently, it also stimulates hydrogen ion excretion exacerbating the alkalosis. Thus, considering our patient with severe hypertension with refractory hypokalaemia and the presence of alkalosis, the possibility of a mineralocorticoid imbalance was raised. Initial abdominal computed tomography (CT), obtained before the biochemical analyses, identified bilateral adrenal adenomas, as confirmed by the radiologist, and supported our theory (Figure 1).

Following the discontinuation of the interfering medication (an aldosterone receptor antagonist), biochemical analysis demonstrated normal plasma renin and aldosterone levels, thereby ruling out the hypothesis of mineralocorticoid excess.

We intensified our study of the endocrine axis with the detection of high cortisol levels, unresponsive to low-dose dexamethasone suppression testing. Following the differential diagnosis, ACTH plasma concentrations where assessed, revealing ACTH-dependent Cushing’s syndrome (Table 1).

**Table 1:** Endocrine and metabolic test results LH, luteinizing hormone; FSH, follicle-stimulating hormone; TSH, thyroid-stimulating hormone; ACTH, adrenocorticotropic hormone ^*^The 'pre' value represents the measure on day 1 prior to the first dose and the 'post' value, the measure on day 3.

Test	Result	Reference values
Serum cortisol (ug/dL)	56.48	5.27-22.45
Salivary cortisol (ng/dL)	>30	0.58-6.54
Urinary cortisol (ug/24h)	5027.1	4.3-176.0
Dexamethasone suppression test (ug/dL)^*^	pre: 47.1; post: 45.1	-
Serum ACTH (pg/dL)	142.3	3.6-60.5
Plasma renin activity (ng/ml/h)	0.7	0.2-1.6
Serum aldosterone (ng/dL)	3.34	1.76-23.2
TSH (mUI/L)	0.78	0.27-4.20
Free T4 (pmol/L)	7.6	12.0-22.0
Prolactin (ng/mL)	5.2	2.8-29.2
FSH (mUI/mL)	9.7	2.5-10.2
LH (mUI/mL)	3.1	1.9-12.5
Estradiol (pg/ml)	28.6	19.5-144
Plasma metanephrine (pg/mL)	71.4	<316.0
Plasma epinephrine (pg/mL)	<15	<84
Plasma norepinephrine (pg/mL)	317.4	<420
Plasma dopamine (pg/mL)	<30	<85

**Figure 2:**
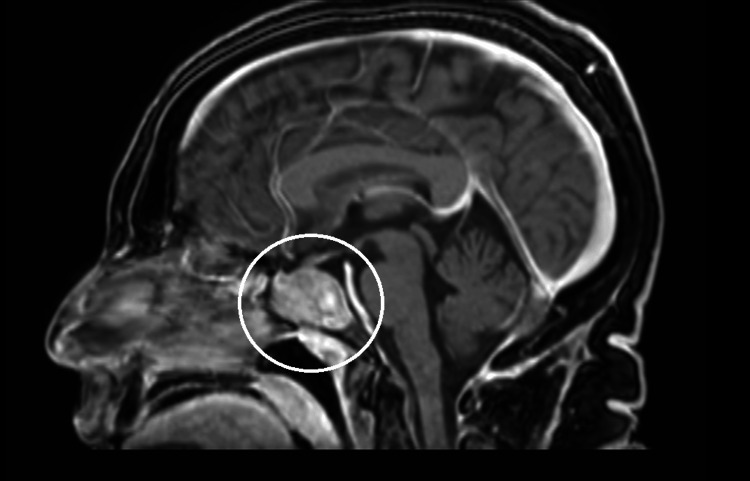
MRI of the pituitary gland, with the circle indicating the macroadenoma of the pituitary gland (sagittal view)

**Figure 3:**
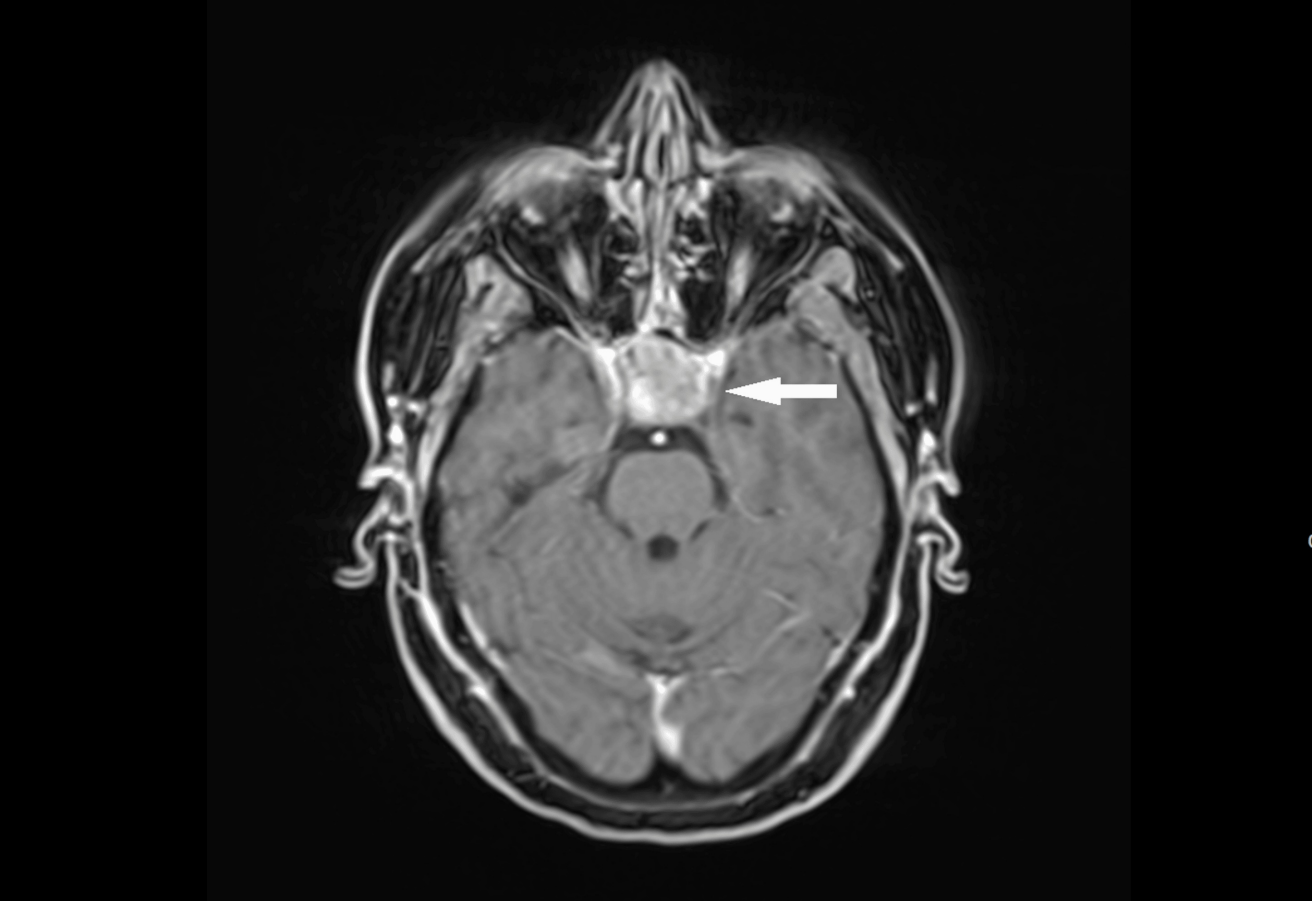
MRI of the pituitary gland, with the arrow indicating the macroadenoma of the pituitary gland

Knowing that the most common cause of increased ACTH is excessive production in the pituitary gland, an imaging examination was performed. MRI of the pituitary revealed a sellar and suprasellar mass consistent with a macroadenoma (Figures 2-3).

CRH testing is used to help differentiate between pituitary and ectopic ACTH overproduction. CRH stimulates the pituitary gland to release ACTH; in patients with Cushing’s disease, CRH administration typically leads to an increase in ACTH and cortisol levels. However, in cases of ectopic ACTH production, CRH will not cause a significant increase in ACTH production because the source of ACTH is not regulated by CRH. The unavailability of CRH testing in our hospital limited further differentiation; nevertheless, clinical and imaging findings supported a diagnosis of pituitary-driven hypercortisolism, or Cushing’s disease. The study of the remaining hormonal axis revealed secondary hypothyroidism, no alterations in sex hormones or prolactin, and the exclusion of other endocrine causes.

The patient was admitted for aggressive management of severe hypokalaemia and blood pressure stabilization. Intravenous administration of potassium chloride and magnesium sulphate was initiated (although magnesium levels were normal), alongside intensified antihypertensive regimens incorporating four drug classes, including high-dose spironolactone. Prior to definitive treatment, it was necessary to stabilize the cortisol levels with metyrapone. Definitive treatment entailed transsphenoidal resection of the pituitary macroadenoma, performed under the Neurosurgery department. Since the surgery was performed at another hospital, we did not have access to the histology report.

Postoperative recovery was marked by substantial improvement in blood pressure control, normalization of serum potassium levels, and gradual weight loss. The patient’s antihypertensive regimen was significantly reduced in complexity, with only one class of drug required. The follow-up in the outpatient Endocrinology Clinic has demonstrated sustained clinical stability, three years after the hospitalization.

## Discussion

Cushing’s disease exemplifies the complex interplay between endocrine dysfunction and the systemic impact of cortisol. Cardiovascular complications are the most prominent and significantly contribute to mortality and morbidity. Elevated cortisol promotes sodium and water retention, increasing blood volume and raising blood pressure, that when sustained is a major risk factor for cardiovascular events, such as stroke, myocardial infarction, and heart failure. Additionally, cortisol’s effects on the vascular system, including promoting inflammation and endothelial dysfunction, contribute to the development of atherosclerosis. The type of obesity induced by cortisol, with abdominal fat accumulation, is also a risk factor for metabolic syndrome, further contributing to cardiovascular complications [3,5,11,12].

Cortisol plays a key role in the regulation of mood and emotional responses, and elevated cortisol levels can impair the function of the hippocampus, which is essential for memory and emotional regulation. Prolonged high cortisol levels can cause hippocampal atrophy and disrupt the balance of neurotransmitters, resulting in depression. In terms of reproductive function, excess cortisol can disrupt the hypothalamic-pituitary-gonadal axis, which is crucial for regulating ovulation and the menstrual cycle. In women, this disruption can lead to anovulation and irregular menstrual cycles, potentially causing infertility. Elevated cortisol also interferes with the production of estrogen and progesterone, further disrupting the menstrual cycle. Cortisol’s negative effects on the thyroid axis, which also plays a role in reproductive health, can contribute to hormonal imbalances, further affecting fertility. Additionally, hypercortisolism impacts bone and muscle health, with severe osteoporosis and muscle atrophy. A frequently underappreciated consequence is the risk for severe infections and psychiatric disorders [3-5].

This case illustrates the importance of integrating clinical, laboratory, and imaging data. Initially, based on the laboratory test abnormalities and hypertension, an examination focused on the adrenal glands was performed, which led to an assumed diagnosis. Although adrenal adenomas were identified, they were likely non-functioning adenomas since there was no biochemical evidence to support their activity. The integration of data helps prevent diagnostic errors. When a more detailed medical history was taken, the symptoms were more suggestive of hypercortisolism. This was confirmed by the laboratory and imaging findings, leading to the diagnosis of ACTH-dependent hypercortisolism, or Cushing's disease, due to a pituitary macroadenoma.

Transsphenoidal surgery is the standard treatment, particularly in cases of a hormonally active tumour or with mass effect. Even though it is a minimally invasive approach, the risk of complications, such as hypopituitarism due to damage to the pituitary gland, cerebrospinal fluid leaks, infections, and visual disturbances due to damage to the optic pathways, needs to be taken into account. When surgery is not feasible or not successful, other therapeutic strategies must be considered. Steroidogenesis inhibitors are used to reduce cortisol production by inhibiting enzymes involved in cortisol synthesis. Pituitary-targeted therapies can be effective in certain patients, particularly those with tumours that secrete ACTH. For patients with persistent or recurrent disease following surgery or in those who are not surgical candidates, pituitary irradiation is an option. In some cases, bilateral adrenalectomy is considered, particularly when other treatments fail, though this requires lifelong hormone replacement therapy [9-10].

Monitoring treatment in Cushing’s syndrome is critical to assess the effectiveness of therapy, detect complications, and evaluate for recurrence or residual disease. Regular follow-ups involve monitoring for resolution of Cushing’s symptoms, such as improvements in hypertension, diabetes, weight, skin changes, and psychological symptoms. Biochemical markers, with 24-hour urinary cortisol, serum cortisol levels and late-night salivary cortisol, are essential in monitoring cortisol levels to determine treatment efficacy. In cases of persistent or recurrent disease, MRI is the modality of choice for detecting pituitary adenomas, while CT or MRI can be used to evaluate the adrenal glands and search for ectopic sources. In the case of our patient, follow-up included regular laboratory tests, which showed biochemical control at the two-month follow-up, consistent with the timings described in literature [9,13], and no evidence of complications such as pituitary insufficiency. Repeat imaging with MRI confirmed a complete removal of the adenoma.

The prognosis in Cushing’s disease depends on timely diagnosis and effective treatment. If left untreated, it is often fatal, mostly due to cardiovascular events, and a delayed intervention can lead to irreversible complications. Conversely, early intervention is crucial to reduce the risk of long-term complications and improve prognosis. However, even after a cure for Cushing’s syndrome, many patients may not see a full normalization of cardiovascular risk factors [12]. This suggests that long-term monitoring and management of these risk factors are crucial for improving life expectancy and quality of life as demonstrated in this case.

## Conclusions

This case highlights the importance of recognizing red flags for diagnosing Cushing’s disease, particularly in patients with resistant hypertension and metabolic disturbances that do not align with typical primary hypertension. Key clinical features, such as moon facies, acanthosis nigricans, and violaceous abdominal striae, should raise suspicion for hypercortisolism. In our case, these symptoms, when combined with severe hypokalaemia and hypertension, prompted a thorough investigation that led to the correct diagnosis of ACTH-dependent Cushing’s syndrome due to a pituitary macroadenoma. This underscores the need for a detailed medical history and careful physical examination in the diagnostic process. The collaboration of multiple medical specialties, that is, endocrinology, neurosurgery, and radiology, was critical in this case. Each discipline played an essential role in diagnosing, managing, and following up on the patient’s condition. Given the complexities in diagnosing rare endocrine disorders like Cushing’s disease, it is imperative that healthcare professionals remain vigilant and adopt a multidisciplinary approach. Future research should focus on improving diagnostic tools, particularly non-invasive tests that can more accurately differentiate between various causes of hypercortisolism, reducing the reliance on more invasive procedures like pituitary surgery. Further studies are also needed to refine management strategies for long-term cardiovascular and metabolic risk reduction following successful treatment of Cushing’s syndrome.
